# Rethinking the Methodological Foundation of Historical Political Science

**DOI:** 10.1007/s41111-021-00200-6

**Published:** 2022-01-08

**Authors:** Qipeng Shi

**Affiliations:** School of International Relations and Diplomacy, Beijing Foreign Studies University, Beijing, 100872 China

**Keywords:** Constructivism, Historical–causal narrative, Historical political science, Naturalism, Regularity theory of causality

## Abstract

The basis of a methodology determines whether a research method can fit the core characteristics of a particular academic tradition, and thus, it is crucial to explore this foundation. Keeping in mind the controversy and progress of the philosophy of social sciences, this paper aims to elaborate on four aspects including the cognitive model, the view of causality, research methods, and analysis techniques, and to establish a more solid methodological basis for historical political science. With respect to the “upstream knowledge” of methodology, both positivism and critical realism underestimate the tremendous difference between the natural world and the social world. This leads to inherent flaws in controlled comparison and causal mechanism analysis. Given the constructiveness of social categories and the complexity of historical circumstances, the cognitive model of constructivism makes it more suitable for researchers to engage in macro-political and social analysis. From the perspective of constructivism, the causality in “storytelling,” i.e., the traditional narrative analysis, is placed as the basis of the regularity theory of causality in this paper, thus forming the historical–causal narrative. The historical–causal narrative focuses on how a research object is shaped and self-shaped in the ontological historical process, and thus ideally suits the disciplinary characteristics of historical political science. Researchers can complete theoretical dialogues, test hypotheses, and further explore the law of causality in logic and evidence, thereby achieving the purpose of “learning from history” in historical political science.

## Introduction

The rise of historical political science has provided a “cardiotonic agent” for the eclipsing historical vision in the field of social sciences. The unique attribute of historical ontology and its functional characteristics in this approach have attracted widespread attention (Yang 2019a, b; Yang and Shi 2020). However, when it comes to specific operation, researchers disagree on what analytical tools should be adopted in historical political science. In the initial stage of historical political science, I used to apply some methodological thinking for this new discipline (Shi 2019). However, with my accumulation of knowledge, and observation of the academic situation over the years, I am increasingly aware of the fact that the positivism has lagged behind the development of historical political science. It is impossible to fully summarize the methodological characteristics of this emerging discipline simply through existing analytic tools such as “sequence,” “context,” “case selection,” and “asymmetric causality.” Reviewing the overall academic development nowadays, one can find that the “causal inference revolution” has already stirred the entire political science community (Morgan and Winship 2015; Pearl and Mackenzie 2018). The explosive growth of experimental methods and the growing momentum of “big data” and experimental method have posed a huge threat to the history-oriented methodologies. In this context, this paper starts with the original meaning of “methodological basis” and attempts to provide a more solid methodological basis for historical political science through a discussion on the “upstream knowledge” of specific research methods.

It is inevitable to discuss “upstream knowledge” that has not been defined in many cases, because it shaped the basic views of researchers on the things that they study and the analysis methods that they adopt. The “basis” of a specific methodology directs and runs through the entire research process. It is a skin, not a sweater (Furlong and Marsh 2010). In the case of historical political science, researchers should be concerned about the methodological basis and analytical techniques as these not only test and lead to the discovery of theories, but also perform the function of “learning from history” in analyzing the social beings. To this end, this paper attempts to build the methodological basis of historical political science based on the cognitive model of constructivism and the regularity theory of causality, while seeking to liberate macro-causal analysis from the naturalistic tradition through historical–causal narrative, thereby providing a possibility to further expand the historical imagination.

## The Methodological Debates About Historical Political Science

The debate over the research methods of historical political science is largely due to the fact that researchers have not reached a consensus on the meaning of “history” in historical political science. I find that there are at least two views that are mutually contradictory, each claiming to be devoted to analyses in historical political science.

I would like to describe the first approach as “taking history as background knowledge.” There are two branches under this approach: the application of general theories to historical evolution, and the analysis of specific historic events via different analytic tools. The analysis of history based on general theories was quite popular in the era of structural functionalism, while researchers with a preference for economics are increasingly favoring the combination of historical data and scientific research methods. Generally, few studies rely solely on statistical data or formal models. By contrast, Western academic circles have witnessed a movement to “recover history” in recent years, which has produced “analytic narratives” and “quantitative historical research”. In analytic narratives, “rational selection and historical narrative are combined to study institutions and the effect of the institutions on political and economic behaviors” (Bates et al. 1998). “Quantitative historical research” claims to “bring the whole process of scientific research methods into the field of history” (Chen 2016). From the perspective of analysis strategies, these researchers stress the importance of including the long-term impact of historic events into the framework of causal identification. This helps to analyze the impact of variables like institutions and concepts, which are highly endogenous and of great theoretical significance (Ma 2018).

Many best sellers in social science also fall into this category, authors of these books have cited extensive historical data only to confirm the a priori hypotheses put forward by them (Acemglu and Robinson 2012; Fukuyama 2014). The role of history has been greatly “dwarfed.” Researchers tend to apply popular theories and thinking to explain historical phenomena instead of forming theories and revising hypotheses through rigorous historical investigations. Therefore, the “scientific” research in many cases is nothing but ideologies underlying complex formulas and tables. This trend is very common in economics, such as the attempt by North et al. to construct “a conceptual framework for interpreting recorded human history” (North et al. 2009). This tradition, which considers history as background material, became popular decades ago with the prevalence of structural functionalism. Since the twenty-first century, along with economic imperialism, it has spread rapidly throughout the entire sphere of political science. With respect to this, even an American political scientist complained that “political scientists commonly draw on history but often do not read actual historians carefully. This limited engagement with historians, and with contextual information more generally, contributes to a loss of historical knowledge that can undermine the validity of quantitative analysis” (Kreuzer 2010).

When history is regarded as background knowledge, its meaning is limited to “what happened in the past.” Yet the definition of “what happened in the past” is rather vague: events that occurred in ancient dynasties and even in the early years of the People’s Republic of China (PRC) can certainly be considered as history, but are the events that happened recently or are still happening, like the Covid-19 pandemic, eligible to become topics of historical political science? Going back to the concept of historical political science itself, we may find that “history” in historical political science does not entirely refer to what happened in the past: in the ontological sense, “history” is not the past, but a continuum of “past-present-future.” In essence, historical political science is the “historical” study of political phenomena, paying particular attention to the context and structure where realistic politics can emerge and evolve, and examining them from a dynamic and long-term analytical perspective.

This leads us to the second point of view on history: history is not only about the past; the historical oriented research means temporally oriented research (Mahoney and Thelen 2015; Fioretos et al. 2016). Not only does time represent the one-dimensional physical movement of life, it also contains the multi-dimensional structure of human social movement. The latter embodies what people call a sense of history and constitutes the dimension through which history can be known and understood. The methodological uniqueness of historical political science lies in its ability to skillfully use the analytical tools constructed around “temporalities” to test and generate theories. It is for this reason that historical political science can be understood as an academic tradition in the methodological sense: it focuses on how time and context play a role in the origin and evolution of ideas, institutions, and behavior that affect political, economic, and social relations (Shi 2019).

This tradition is often referred to as “macro-causal analysis” or “comparative-historical analysis” in Western political science circles, and its academic origins can be traced back to the founders of social sciences like Marx, Tocqueville, and Weber. Its contemporary disciplinary foundation was officially established thanks to the publication of *Social Origins of Dictatorship and Democracy* in 1966. The book, which took the author Barington Moore a decade to complete, outlines three paths to modernization through detailed analyses of different cases. Moore proposes a structuralist “meta-theory”: the theory opposes the interpretation of macro-social phenomena based on cultures, values or personal preferences; on the contrary, it advocates that researchers should pay more attention to the objective relationship between organizations and society. It must be noted that Moore’s research shows high sensitivity to history, with comparative research, induction, and causal interpretation being presented in a complex and dialectical manner. The approaches of macro-historical analysis followed by Moore, historical interpretation of Reinhard Bendix and Edward P. Thompson, and universal theory modeling used by S. N. Eisenstadt and Immanuel Wallerstein, constituted a rebellion against the mainstream Parsons’ theory at the time. This ushered in the first historical turn of American social sciences after World War II.

During the development of the discipline over nearly half of a century, Moore’s followers have introduced two rounds of methodologies centered on temporal analysis, the first brought in by Theda Skocpol and the second by James Mahoney (Shi 2019). The methodological characteristics featured in the two rounds have endowed the research methods of historical political science with the following characteristics:

1. Time matters. Timing, sequence, tempo, and rate or duration of occurrence of events, as well as cumulative changes or equilibrium in the historical process, all have a significant impact on the final result.
2. Context matters. The formation of a specific structure, institution, and concept depends on the natural environment, cultural atmosphere, world system, and power network in which it is located.
3. Case-based research, wherein researchers test or propose theories through case analysis and comparison.
4. Configurational and asymmetric causality, which refer to “one cause and multiple effects,” “multiple causes and one effect,” and “differences in the explanation of failure and of success.”
5. A concept is hierarchical and consists of many different dimensions.

Based on the perspective of knowledge accumulation, one can see that the contemporary analytical tools formed around the temporality are becoming increasingly refined. This is a new change in the academic environment. For instance, in sociology, researchers have gradually moved away from the “formation of nation-states and the rise of capitalism” that traditional historical sociology was concerned with, and abandoned grand narrative methods. Historical sociology has ushered in what is referred to as the “third wave,” the core of which is the rise of culturalism and increased attention to the interpretation of individual behavior (Adams et al. 2005). In political science, quantitative research, supported by new technologies, has developed by leaps and bounds, and quantitative researchers have shown the ambition to unify the design of social science research with their own logic (King et al. 1994). Social researchers engaged in historical analysis are facing unprecedented challenges in the whirlpool of the “methodological revolution,” coupled with the emergence of big data and experimental methods in recent years.

Researchers have offered many insights in the field of methodology towards addressing this challenge. Overall, however, the historical turn since the twenty-first century has lost the ambition and vitality shown by the historical turn in social sciences that occurred in the middle of the twentieth century. Even the current qualitative research camp has become more conservative when compared to the beginning of the twenty-first century (Shi 2020). In an interview, Mahoney explained that comparative historical research has matured, and “when a research approach matures, it will become more or less forward-looking and lose some passion” (Li 2018). This suggests that history-oriented social science research must accept the fate of “conventional sciences” as it becomes increasingly specialized: researchers' visions are greatly restricted and paradigm changes are seriously hindered, which inevitably leads to increasingly rigid scientific research. On the other hand, in fields in which scientists have had a paradigm-guided focus, conventional sciences have ensured full and complete information, as well as the observation-theory consistency accuracy that cannot be achieved by any other method (Kuhn 1962). If historical political science uses this as a methodological basis without thinking about it, it may face the dilemma that “once it is introduced, it is already old.”

## The Poverty of Scientism and the Dilemma of Positivism

Historical political science is facing a situation that is worthy of vigilance but has not attracted enough attention: many methodological researchers in today's political science circle are so obsessed with the illusion of naturalism and the causal idea of positivism as they seek to make historical analysis more “scientific,” that their historical imagination has been eroded. Here, positivism is equated with logical positivism, which advocates that causal explanation is the logical result of combining the general laws with specific cases in social life. Using the deductive nomological explanation (D-N model), researchers have found universal laws that exist in the social world as those in the natural world, and with these laws, they can predict results. Mainstream social science research methods guided by this philosophical paradigm have been viewed with skepticism as the blind pursuit of refined methods and rigorous logic has caused scholars to stop addressing major issues in reality (Shapiro 2007). Unfortunately, positivism has a similarly huge impact on the history-oriented political and social analyses. In the following section, I will explain why positivism-based controlled comparison and mixed methods can hardly serve as the methodological basis of historical political science.

### The Illusion of Controlled Comparison

Controlled comparison is based on counterfactual causality, which reflects how contemporary positivism perceives science (Woodward 2003; Rubin 2005). In specific instances, experimental methods based on counterfactual logic can effectively identify the cause, establish the direction of the causal relationship, and rule out false relationships through human manipulation and intervention of key explanatory variables. To put it simply, “experimental methods” refer to the research methods in which the researcher manipulates the intervention of the subject, whether it be human or things, to observe the causal relationship (Cox and Reid 2000). There are two mainstream views currently: one, social science experiments have an absolute advantage in terms of internal validity and can even be regarded as the gold standard for causal inference (Holland 1986). This is because experiments can better control the influence of confounding factors and cleanly isolate causal effects. In other words, differences or changes in results, which are observed in experiments, can be attributed to different interventions. Arend Lijphart points out that “in terms of their scientific explanatory power, experiments are almost the most ideal method, but unfortunately, due to practical and ethical obstacles, this method is seldom used in political science” (Lijphart 1971). As an alternative supplement, quantitative statistics, which are based on multiple regression analysis, examine whether the “probable cause” affects the “possible outcome” by controlling other influencing factors. Statistical analysis uses partial correlation to conveniently handle the issue of control, while qualitative researchers use controlled comparison to address the problem of “too many variables and too few cases.”

Controlled comparison refers to the control of non-explanatory factors in the process of case comparison, to achieve the “quasi-experimental” state to the greatest possible extent. Many books on contemporary comparative political studies analyze several countries in a specific region, which is in fact control of factors such as geographic environment, resource endowments, and cultural traditions. Methodological thinking based on controlled comparison (Przeworski and Teune 1970; Skocpol and Somers 1980; Brady and Collier 2010) has grown to become the mainstay of contemporary small-sample analysis, and has provided powerful analysis tools to understand major issues like the outbreak of revolutions, the influence of social capital, the formation of national capabilities, ethnic violence, and social mobilization. It is for this reason that some scholars propose that if the prevailing variables and mechanisms can be chosen in controlled comparison to explain the significantly different consequences, the method will gain both internal and external validity (Slater and Ziblatt 2013).

Nevertheless, the criticism about controlled comparison from the era of *States and Social Revolutions* lingers till this day. Over the past three decades, “omitted causes” and “measurement errors” have troubled researchers who are engaged in small-sample comparisons (King et al. 1994; Goldthorpe 1997; Lieberson 1994). Qualitative researchers are also sharply divided among themselves. Some believe that the proponents of controlled comparison are overconfident in their ability to discern complex causal relationships while underestimating the impact of interactions. Additionally, controlled comparison may be far from being as predictable as it claims, and it may cause researchers to ignore potential causality. Therefore, most methodological researchers acknowledge that the approach is a “weak” method of causal inference, “unless one abandons cross-case analyses entirely for large-N methods such as regression analysis and QCA, there is no way to avoid cross-case inferences in case studies” (Rohlfing 2012).

The fragile methodological basis has shattered the hope: controlled comparison has established itself in a cognitive model system where it is always at a disadvantage. In the cognitive model of positivism, researchers have made efforts to approach the “quasi-experimental state” using scope conditions, and negative and semi-negative cases to limit the time and space dimensions of macro-causal analysis (Mahoney and Goertz 2004; Ye and Shiping 2019). Despite this, experimental methods are always stronger than large-sample statistics and small-case comparisons in terms of the validity of causal inference. Specifically, the “control” in controlled comparison is far from reaching the standards of experimental methods, and the “quasi-experimental state” is nothing but wishful thinking of the researchers. Just as identical twins have extremely different behavior patterns, macro-political and social phenomena are far from being as easy to control as scholars imagine (Diamond and Robinson 2011). Natural experiments that have emerged in recent years are nothing but applications of the “most similar system design.” Placing the task of causal inference on controlled comparison cannot achieve the strong causality anticipated by researchers, and it may also greatly narrow their space for topic options—in the view of controlled comparison, many meaningful research topics will be abandoned for not being “scientific” enough.

So, how should comparative research be conducted? In my opinion, historical political science should discard the practice of achieving causal inference through “comparison” and seek a more flexible comparison strategy. “Comparison” is not an exhaustive search for causes of results, but an effort to illuminate certain aspects of the complex social situation (Lichterman and Reed 2014). An approach called “comparative intuition” is worthy of attention. Under this approach, things that otherwise seem impossible to compare can be understood and analyzed within the same framework. For example, a French president can be compared with an African chief, because through such comparison, researchers can understand how people in power mobilize society, threaten as part of an alliance, fight opponents, implement changes, and maintain the status quo (Boswell et al. 2019). When “comparison” is no longer about “seeking cause and effect,” the old approach will certainly be reinvigorated:

“It is important to recognize that comparison is not a method or even an academic technique; rather, it is a discursive strategy. There are a few important points to bear in mind when one wants to make a comparison. First of all, one has to decide, in any given work, whether one is mainly trying to identify similarities or differences. It is very difficult, for example, to say, let alone prove, that Japan and China or Korea are basically similar or basically different. Either is possible depending on one’s view, one’s framework, and the conclusions towards which one intends to move” (Anderson 2016).

Benedict Anderson reminds us that the perspective, analytical framework, and theoretical dialogue of scholars in comparative research are very important. This is similar to Skocpol’s point of view: “For the comparative method alone cannot define the phenomenon to be studied. It cannot select appropriate units of analysis or say which historical cases should be studied. Nor can it provide the causal hypotheses to be explored. All of these must come from the macro-sociological imagination, informed by the theoretical debates of the day, and sensitive to the patterns of evidence for sets of historical cases” (Skocpol 1979). Therefore, the key to macro-causal analysis lies not in subtle methods, but in the profound interactions among macro-historical imagination, social reality, and existing theories. With the help of macro-historical imagination, historical political science will be able to get rid of the stereotype that “seeking differences is more advantageous than seeking common ground in constructing contingent associations.” On the contrary, research on similarities can better “face the fact that common political phenomena will overturn popular ideological theories and may question the legitimacy of Western political systems” (Yang 2016a, b). From Aristotle to Montesquieu to Tocqueville, from Marx to Weber, classical comparative research appears to have no intention of realizing the so-called scientific causal inference under strict control; rather, “comparison” understands the important perspective of how diverse the real world is. That is why it is argued that “all social sciences are comparative, it’s just that different people have different analysis theories and different dimensions of comparison” (Wang 1987).

### The Double Failure of the Mixed Method

The mixed method has also become a hot topic in the field of methodology in recent years. Mixed method means that researchers mix or combine quantitative and qualitative techniques in their research (Johnson and Onwuegbuzie 2004). Generally, quantitative research can realize the generalization of the theory, while qualitative research can unearth the causal mechanism in cases. Therefore, the purpose of the mixed method is to achieve the complementary advantages of these two techniques in one research design, and then to confirm the same conclusion through different research methods.

The mixed method demonstrates the wish of methodological eclectics that “the more methods, the better.” Yet it must be admitted that the analytical effectiveness of different research methods varies, and this is mainly due to the topic under analysis. The combination of different methods sometimes even undermines their respective advantages. For example, in the combination of formal model and case comparison, rational selectionism is at the risk of having its own explanatory power undermined while failing to make full use of the advantages of comparison (Mahoney 2000). That also explains why “analytic narrative,” which is a mixture of rational selection and historical analysis and advocated by a group of heavyweight scholars, has not been effectively promoted over the past two decades. In the current stage, most mixed methods only show how different logics are working in their own way. Therefore, people in favor of true pluralistic methods advocate the integration of different methods into a unified causal inference (Seawright 2016).

More importantly, advocates of mixed methods have blurred the difference between “method” and “methodology”. “Methods” are particular tools and techniques for analyzing the social world, whereas the “methodology” refers to a family of methods that share similar foundational ontological and epistemological assumptions (Beach and Kaas 2020). It is feasible to mix different research methods, but not methodologies as there may be conflicts between epistemology and ontology, which are the basis of methodology (Ahmed and Sil 2009; Chatterjee 2011). Unfortunately, many contemporary popular mixed methods, such as nested analysis, try to synthesize methodologies of different logics.

Take the theoretical argument over landlord class vs. democracy as an example. Both structuralism and rational choice theories believe that the landlord class is a huge obstacle to democracy. However, the case of the Philippines has shown that a powerful landlord class can coexist with free democracy. In this case, variable-oriented researchers regard the Philippines as an “outlier,” which has no impact on the validity of the existing theory; while case-oriented researchers see that the case of the Philippines suggests flaws in the existing theory and that they should revise the theory by enriching the knowledge system and deepening our understanding of the complex world with new ideas such as “feudal democracy” (He 2020). The difference between the two analytical perspectives stems from the difference in ontology, which constitutes the methodological basis: quantitative researchers uphold the ontological assumption of probabilism, believing that the real world is full of randomness and what is shown between cause and effect is only a trend, which is not necessarily true in individual cases. Therefore, the few cases within the credible interval that are not consistent with theoretical expectations do not weaken the causal effect of the theory. On the contrary, qualitative researchers hold determinism, and they believe that the real world is deterministic—“there is at any instant exactly one physically possible future” (Inwagen 1986), and abnormal cases serve as a cornerstone for theories to be revised.

It can be inferred that the ontological conflict has led to logical inconsistency in the mixing of different methodologies: researchers cannot make a case to be both an “outlier” within the scope permitted by the theory and as “key evidence” to subvert the existing theory. The only possible solution is methodological monism, that is, there is only one mode of causal inference in social sciences, as claimed in *Designing Social Inquiry* (King et al. 1994). In other words, the credibility of mixed research is premised on a single methodological basis, which remains positivism, in most cases. In most mixed research designs, case studies merely enrich the overall trend derived from statistics, so essentially, such research is still a variable-oriented methodology.

In summary, it is not difficult to see that causal analysis, which is dedicated to exploring big outcomes and large processes, has been shrouded in the shadow of positivism. Under a high degree of “methodological self-consciousness,” “the entanglement of quantitative and qualitative methods in analytical tools, the difference between nation-states and sub-states at the analysis level, the tangle of cross-case and inter-case comparisons in terms of case number, the swing of comparison and temporalities in the focus of analysis, and the demarcation of political science and sociology in the disciplinary division” only lead to the deviation and erosion of historical imagination (Ying 2021). It is worth mentioning that the philosophical foundation of science on which positivism relies, i.e., the Newtonian mechanics theory since the nineteenth century, has gone well past its prime in the field of natural science. In the middle of the twentieth century, when the basic framework of Western mainstream political science was finally established, although the internal Newtonian world view of physics had come under fire, it impacted social sciences to the greatest extent (Wallerstein 2001). Many Newtonian scientific views, including the objectivity of the world, the absoluteness of time and space, the existence of universal laws in the objective world, and individual rationality, still prevail in social sciences. The resulting “methodological self-consciousness” places historical political science on a fragile foundation that is about to collapse. It is imperative that historical political science get rid of the prejudices and shackles brought about by positivism, to seek a methodological basis.

## The Transcendence and Dilemma of the Causal Mechanism Method

Obviously, positivism, on which controlled comparison and mixed method are based, is unable to provide a solid methodological basis for macro-political and social analysis. Looking back at the history of the philosophy of science, one may find that positivist epistemology, which is popular in the contemporary world, was fundamentally subverted as a philosophy as early as the middle and late twentieth century. In other words, philosophy of science has already moved from positivism to post-positivism. Although post-positivist philosophy of science is divided, in general, researchers have reached an agreement on two things: one, positivism in the past seriously misinterpreted the true intentions of natural science, and the prevailing logical positivism has never established a true correspondence with empirical research in the history of science; two, the separation of value and fact and the so-called “value neutrality” are impossible. Facts are never transparent, and any description based on a phenomenon contains a certain theory (Zhu 2021). In short, researchers need to provide a new causal explanation model in addition to positivism.

In the 1970s when positivism was severely criticized by the entire philosophy circle, the British philosopher Roy Bhaskar came up with “critical realism,” which arose as a force not to be overlooked. The most important philosophical proposition of critical realism is stratified ontology: human knowledge is classified into two groups, the inaccessible and the accessible. All scientific research objects belong to the inaccessible group, and all scientific knowledge and theories or discourses, which can be modified or synthesized, belong to the accessible group (Sayer 2000). Therefore, unlike positivists or post-positivists who regard the existence of experience or the existence of ideas as the essential characteristic of reality, critical realism emphasizes the existence of ontological reality independent of human cognition (Joseph and Wright 2010). Moreover, the real research object is composed of three overlapping domains that go from the shallower to the deeper, namely the empirical, the actual, and the real. Among them, what constitute the essential characteristic of the world are the causal power, causal mechanism, and deep structure that exist in reality. “Mechanism” is “the way in which things work.” There are various mechanisms in and among things, and it is a major task of scientific researchers to explore various mechanisms in reality (Bhaskar 1997).

Critical realism has developed amidst controversies among different schools of positivism and post-positivism, and rapidly extended from philosophy of science to philosophy of social sciences. It has offered completely different insights in terms of causality, structure–agent relationship, the definition of “explanation,” and the understanding of “knowledge” and “value” (Gorski 2013). In the late twentieth century, some historical sociologist began to pay attention to the philosophical propositions of critical realism. George Steinmetz believes that most social science researchers engaged in historical analysis have demonstrated the characteristics of critical realism to varying degrees (Steinmetz 1998); Philip G. Gorski notes that Skocpol’s *States and Social Revolutions* is an excellent presentation of critical realism (Gorski 2004). The exploration of “deep structure” and “causal power” in critical realism coincides with the purpose of historical political science. It is for this reason that Professor Zhu Yunhan believes that “critical realism can be used as a new major philosophical theory and program to guide social sciences or future historical political science. It has a strong affinity with many pioneers of historical sociology and historical structure analysis (including Marx) and their intellectual activities in the past” (Zhu 2020).

As an alternative, critical realism has indeed reshaped the ecology of social science research. More and more scholars have begun to shift from causal effects to causal mechanism. Although their definition of mechanism varies greatly, the wide application of “causal mechanism” or “causal power” undoubtedly demonstrates the powerful influence of critical realism. Today, realists have roughly agreed upon the following aspects pertaining to the definition of causal mechanism: one, mechanism is the real process of propelling or preventing changes in the real social system; two, the interaction of mechanism and factors drives the result of the social system, which indicates that the two are interdependent (Tang 2018). In other words, mechanism is a real entity. Unlike positivists who regard experimental methods as the holy grail of causal inference, realists believe that discovering a mechanism is the gold standard for establishing and explaining causal connections (Glennan 2009).

Notwithstanding, whether critical realism can serve as the methodological basis of historical political science is still open to question. Here, I would like to raise a prerequisite question: Does the causal mechanism really exist as claimed by the causal view of the mechanism theory?

In the view of critical realists like Bhaskar, despite the difference between the “social world” and the “natural world,” researchers can study their similarities by exploring the deep structure and causal mechanism. However, in my opinion, realists have greatly underestimated the difference between these “two worlds.” Critical realists often use examples of the natural world, such as the process of gaining and losing electrons in redox reactions, to make an analogy with the operation of the causal mechanism. Yet this analogy is not appropriate for social science research. The most crucial point is that the categories of social sciences are not simple reflections of social reality, and they are not constant in nature. Let’s take a look at the following three categories, which show the vast differences between research objects of the natural world and the social world:

The first kind of the category includes things such as atoms, electrons, quarks, wave packets, and nuclear decay, which really exist as objective entities, and they all have an attribute that can be called “essence”: the essential attribute of atoms is determined by the nucleus and extranuclear electrons; the same atoms form different substances in different arrangements (such as diamond and graphite); and nuclear decay and redox reactions are real processes with specific “causal powers,” etc. Such phenomena exist in the basic research of natural sciences, and they are included in the same category because they have the same essential attribute. Ontologically, natural kinds are prior to human beings and their activities and cognitions (Browning 1978; Hacking 1991).

The second kind of the category includes things such as elephant, green, male, hill, and bone hyperplasia, which have some of the essential attributes, as well as people’s cognition. For example, “green” and “red” are obviously two different colors, but the reason why we say “the five-star red flag is red” is just that the flag reflects the light of a wavelength between 520 and 570 nm. For cats, the five-star red flag is definitely not red (cats cannot tell red from green as they are “color-blind” in the human sense of the term). Therefore, the definition of color depends not only on the specific wavelength, but also on people’s conceptualization. For another example, we can clearly distinguish landforms like “hills” and “valleys,” but we can hardly give a unified judgment on what a “hill” is. Therefore, although such things exist objectively, people's understanding of them depends on their “essential attribute” and “individual cognition.”

The third kind of the category includes things such as state, democracy, economic growth, and social revolution, about which social science researchers are concerned. Such things exist in the social world, and the core feature that distinguishes them from those in the first two categories is their “constructiveness.” For example, both Athens that existed thousands of years ago and today’s America are called “democratic regimes.” The reason is not that the two regimes are identical, but that researchers “consider” them as “democratic.” George Lakoff, a leading figure in cognitive linguistics, summarizes research in different disciplines spanning more than two decades and finds that specific “categories” do not gain meaning from their correspondence with natural entities; instead, their meaning exists in the cognitive model of constructivist thinking (Lakoff 1990). Ontologically, human kinds depend on human brains, the meaning of the human kind is dependent on larger matrix of categories and meanings (Geertz 1973; Taylor 1985). Therefore, the category of social sciences is not a direct reflection of social reality, nor is there an “essence” that determines its attributes—it is the product of the interaction between the inner “mind” of man and the outer “reality.”

The causal power school is based on the substantive causal mechanism with causal power, but when the category of things in the social world does not have an essential attribute, that connection no longer exists. Therefore, although the term “mechanism” has become more popular in social science research and researchers can find connections and test theories in the course of events, they can never find a constant force connecting different categories, like physicists. Meanwhile, the constructiveness of the social category can also help us understand the impoverishment of positivism as it adheres to essentialism, just like realism. For example, when analyzing democracy and economic growth, researchers first assume that both “democracy” and “economic growth” have a fixed and single attribute, and has certain “causal powers” that have not yet been discovered. The purpose is to explore the connectivity mechanism or the overall trend that it reveals through different methods. However, researchers have neglected that the political systems of different countries may exhibit completely different political logics, and the causes of economic growth may also be vastly different. Therefore, when completely different social realities are included in a category constructed by people, it is understandable that there can hardly be a stable connection amongst them.

To sum up, although critical realism has achieved great progress when compared to positivism, it underestimates the vast difference between natural science and social science, which leaves researchers stuck in the rut of naturalism. Scholars in favor of the naturalistic beliefs simplify the social world to a concrete representation of interests, power, or normative beliefs. “When they think that politics is about interests, power, or beliefs, they start to lose interest in and sensitivity to the real world of life, as well as the academic quality to grasp the meaning of life” (Luo 2019). That poses a significant damage to historical analysis, because it puts theoretical logic above historical logic. For the empirical world studied by man, “there are portions of the real world, objective facts in the world that are only facts by human agreement. In a sense, there are things that exist only because we believe them to exist” (Searle 1997). These “facts that can only be achieved by unity” exist extensively in the social world under social science research. In other words, social science research is about exploring the material world in the conceptual world. To accommodate the concept of the social world, I believe that historical political science should be based on constructivism, or more exactly, on “scientific constructivism,” which is the philosophical basis of social sciences (Mahoney 2021). Specifically, the cognitive model of historical political science is constructivist, and it holds that the research category of social sciences is the product of the interaction between social reality and the human mind. In this view, the empirical world faced by social sciences is derived from social construction rather than exogenous objective existence (Berger and Luckmann 1967; Spiegel 2005). At the same time, historical political science sticks to the “scientific” approach in the process of empirical analysis and follows the general program of scientific research, that is, “science intends to form theories and to test these theories through empirical observation” (Lakatos et al. 1980).

The constructive nature of the social category requires researchers to “memorize and analyze the construction process of the empirical world (Erfahrungswelt) as an overall structure composed of different arrangements of these meanings” (Schutz). Therefore, history-oriented social science research has turned to focus on the world of meaning constructed by the interweaving of the real world and theoretical concepts. All research objects are the products of historical process. What is important is not how the specific parameters of the research objects have changed, but how their “meaning” has changed.

## Historical Causal Narrative

After identifying the new “upstream knowledge” for the research methods of historical political science, I proceed to look for an alternative to positivism and realism at the methodological level. I prefer a historical analysis model which requires no elaborate efforts and where the overall situation of macro-political and social phenomena is explored through “narrative” or “storytelling.” Throughout the development of social sciences, the use of “storytelling” to analyze political and social phenomena and the world of meaning originates from the idea generated by the “narrative turn” since the 1960s, and the “story” has become the starting point of theoretical research on topics like identity and feminism. It is on this basis that I bring it into the analysis of macro-political and social phenomena. In the days when *States and Social Revolutions* sparked fierce debates, William H. Sewell argued that Skocpol’s book is convincing, not because of the rigid variable patterns, but because she puts a lot of emphasis on “the interaction of situational development between decisive processes that were originally irrelevant” (Sewell 2005). Under the analytic tradition known as “plural causal narrative” or “eventual time,” history can be seen as a “sequence” composed of “events,” and causal analysis is not restricted to the simple correlation between the independent variable and the dependent variable, but extended to the connected events, interactive actions, and historical fluidity (Abbott 2016). On this basis, scholars of the interpretation school in contemporary social sciences call the causal system, which rests on variable, control, and mechanism, as the “forcing-cause,” arguing that such an understanding of causality is based on the interpretation of pre-defined objects and pre-corresponding results. Causal analysis has therefore turned to “tracing these objects moving towards the result and their interactions,” neglecting that the research objects themselves are the product of historical process. The change in the content, category, and characteristic of the research object is not one in its physical parameter, but one in the “meaning” of the parameter itself. Accordingly, a causal approach called “formation stories” is raised. It stresses that the purpose of research is to explain how social existence forms a specific state in the process of self-shaping and being shaped. “Causality” is about revealing the historical process by which a certain social existence is formed (Hirschman and Reed 2014).

The preference for the historical narrative or “storytelling” reflects the efforts of scholars who are tired of methodological debates to “return to common sense”: causality is based on credibility, and logical and convincing stories are those that can excite readers and make more sense. Even though the “storytelling” approach has become quite popular in historical sociological analysis after the cultural turn, it has been questioned in today’s political science world. The traditional view is that if researchers focus on stories or texts, social sciences will be reduced to inferior disciplines. “Narrative analysis is essentially a ‘hodgepodge.’ The disorganized texts, the arbitrary observations, and the entangled logic make the inferences untenable” (Bain 1935). The main reason is that narrative analysis offers no “causality.” In positivist tradition, which plays a dominant role today, counterfactuals are the “gold standard” of causal inference. The seemingly disorganized historical narratives obviously cannot be “counterfactual” through precise “control.” Despite realists’ emphasis on the importance of causal mechanism, historical narratives are unable to clearly show what kind of “causal power” plays a role in the deep structure.

Here is not the place to systematically comment on the two views held by the causal power school and the counterfactual school of causality. But what is worthy of attention for researchers is that the dispute between the two has shown that people have more than one perception of the causality. Here, I would like to make a bold move by placing narrative analysis, which centers on “storytelling,” in the context of an older but controversial view of causality, namely the regularity theory of causality created by David Hume (Hume 1740/1978). It suggests that “cause” is a combination of three conditions: temporal priority, spatiotemporal contact, and constant conjunction (Psillos 2002). Followers of this tradition tend to focus on philosophical discussions (Baumgartner 2013), but I find that the regularity theory fits well with the causal narrative of historical political analysis.

First, “temporal priority” means that researchers should be very sensitive to the sequence of events in the historical process. Such sensitivity has a dual purpose: on the one hand, it provides the historical narrative with clear historical coordinates, which can help researchers to conduct narrative analysis according to the sequence of events; on the other hand, “temporal priority” means that “any event or process is surrounded by its position in time, its position in the sequence of occurrence, and its interaction with various processes exhibiting at different speeds” (Pierson 2004). Therefore, it is necessary for researchers engaged in macropolitical and macrosocial analysis to understand the significant impact of the sequence on the big outcome and large process.

Second, the “spatiotemporal contact” between cause and effect in macro social sciences is often embodied as “indirect proximity,” that is, event X leads to result Y through a series of events (Psillos 2002). The purpose of the historical narrative is to explore the complex causes and evolution of specific results. To that end, comparative history researchers often focus on the overall causal chain composed of events, not just the relationship between cause and effect (George and Bennett 2005; Falleti and Lynch 2009; Beach and Pederson 2019). One must note that it is only the regularity theory of causality that considers the “mechanism” connecting cause and effect as part of the causal relationship. The causal power school regards causal mechanism as a generative force and an unobserved entity instead of interconnected events; counterfactual causality only looks at the functional relationship between independent variables and dependent variables: it just explores the causal relationship without identifying the causal mechanism or treats the mechanism just as an intermediary variable.

Third, “constant conjunction” has been criticized for a long time. A major part in which mechanism theorists surpass their positivist counterparts is that they regard causality as a constant regularity. But in my opinion, many criticisms are more often directed at the covering law model, a branch of Hume’s theory in the twentieth century (Hempel 1966). Such criticisms are mostly pertinent, because the covering law model emphasizes that the universal characteristics of regular forms are evidence of causal knowledge, and “a statement of its meaning does not require reference to any one particular object or spatiotemporal location” (Hempel 1965). The regularity theory is nothing like the covering law model, which simply treats causality as prediction and ignores the way things work. On the contrary, as mentioned in the prior paragraph, the “spatiotemporal contact” prompts researchers to pay attention to the specific connection between cause and structure. Therefore, the “regularity” between cause and effect in macro-political and social analysis is more often reflected in the fact that the cause is a sufficient condition, a necessary condition, a necessary and sufficient condition, an insufficient, but necessary part of an unnecessary but sufficient condition (INUS condition), or a sufficient but unnecessary part of an insufficient but necessary condition (SUIN condition) for the result (Mahoney, Kimball and Koivu 2009). The regularity theory does not advocate absolute constant conjunction. We can find in Hume’s discussion that his “constant conjunction” refers to the connection in a like situation. In constructivism, the causal chain composed of events is a constructed category, and when the meaning of the category changes, the conjunction that was once regarded as constant, changes accordingly. That coincides with many methodological studies of comparative history (Mahoney and Goertz 2004; Falleti and Lynch 2009). Jack A. Goldstone makes a visual distinction between “the covering law” and “discovering the law of history in contingency”:

“… identifying similar sequences of events in different historical contexts is not the same as looking for general laws independent from the historical context. Rather, identifying such sequences is more like a geologist’s activity in mapping different regions and discovering similar fossils in similar layers of rocks in different places. The geologist will then hypothesize that a common process occurred in both places and will attempt to carefully reconstruct what that process was. But this process is not a ‘law’ in the same sense as the law of gravity” (Goldstone 2016).

It should be noted that although I advocate the return of Hume’s view of causality, it does not mean that the view is applicable to all fields. This paper emphasizes that different views of causality have their own advantages in different disciplines or research fields. The mechanism theory of causality is applicable to the basic fields of natural science, such as the exploration of universal gravitation, the movement of celestial bodies, and chemical reactions. Counterfactual causality is more suitable for double-blind experiments in psychology and drugs. The significant rise of randomized controlled trials in recent years has shown an increasing prominence of counterfactual logic in the design of social science research, but whether it will become the “gold standard” is doubtful (Cartwright 2007). The reason why the regularity theory of causality is suitable for the analysis of macropolitical and macrosocial phenomena is closely related to the constructiveness of the social world and the complexity of history. On the one hand, complex history and reality cannot really put things under control, making the counterfactual causality useless; on the other hand, things studied in social sciences, i.e., the “humanly constructed, mind-dependent categories” do not have the constant causal power to “promote things to work.” In the cognitive model of the regularity theory, causality is regarded as a way of cognition in which people describe the regularities formed by the constructed social categories in chronological order.

In summary, I would like to call such a narrative analysis, which is based on the epistemology of scientific constructivism and the regularity theory of causality, as the historical-causal narrative. It effectively bridges “description” and “explanation” in exploring the meaning of the conceptual world: the former is dedicated to describing certain aspects of the complex world, and its purpose is to explain “what” a phenomenon or a group of phenomena is; and the latter tries to explain “why,” assuming that one factor or a group of factors cause the change of the result or produce a specific result (Gerring 2012). In my opinion, such a new analytical approach that differs from positivism and realism in terms of epistemology and from counterfactual tradition or causal mechanism in terms of causality, is sufficient to provide historical political science with a more solid methodological basis.

First and foremost, historical–causal narrative is eligible to analyze “history” with ontological significance, and this sets historical political science apart from historical sociology. The “history” in historical political science is not a simple historical outlook, but an ontological “existence.” This ontological attribute makes historical political science naturally reject the complex historical studies where abstract theories, transcendental concepts, and local knowledge are used for analysis. As mentioned earlier, the core purpose of historical-causal narrative is not to observe the relationships among the categories that have been given a priori specific action logic and causal power, which is very common in positivism, but to explore the historical process in which a specific social existence is formed. The evolution of these social existences is “generated within the historical mechanism that transcends the individual and is formed by institutions and the historical process where the institutions are operated” (Yang 2019a, b). The “continuation” and “generation” of the ontological “social existence” of “history” is analyzed through the narrative.

Secondly, since historical political science deals with the generation and evolution of the ontological existence of history, its research naturally focuses on major issues of the times rather than the relationship between variables. Historical political science has always paid attention to what C. Wright Mills calls problems “of direct relevance to urgent public issues and insistent human troubles” (Mills 2000). Understanding these problems is the key to understanding the actual world. Larry Laudan, a well-known researcher on philosophy of science, argues that science in essence is the activity of “problem solving”: good theories are not valid defenses within the existing epistemological framework, but those which are able to resolve ambiguity, to reduce irregularity to uniformity, and to show that what happens is somehow intelligible and predictable (Laudan 1978). However, the dominant positivist research methods have forced scholars to shift the focus of analysis from “problem solving” to “the validity of existing theories.” Researchers should discuss issues such as causes of different types of government and the formation of different levels of national governance capabilities. Instead, restricted by the mainstream cognitive models, researchers have made more efforts to prove or falsify the relationship among specific categories using cutting-edge research tools. Issues studied by them include whether economic growth can promote democratization, whether war made states, and whether natural resources hinder democratic transformation. For researchers engaged in the state of China, they are restricted to Tilly’s proposition of “war-made states” if they focus on relationship; but if they focus on result, what lies at the center of China’s state issue is the “Great Unification (Dayitong).” Large-sample statistics, emerging experimental methods, and big data may prove inadequate for the generation and evolution of the social existence of “great unification.” Here, historical-causal narrative focusing on complex historical changes and plural causal relationships is undoubtedly a very powerful analytical tool.

Third, historical–causal narrative fits in with the functional appeal of “learning from history” in historical political science. As we know, another prominent feature of historical political science is reflected in its function of “seeking good governance,” which is “to be achieved by paralleling history with reality”. This requires researchers to get rid of the humanistic tradition in their observation of history, in which attention is paid only to the particularity of cases and the macro context is ignored. “Learning from history” refers to the summary of regularity, and this is exactly what historical–causal narrative, which is based on “constant conjunction,” possesses. The pursuit of regularity in historical–causal narrative is reflected in the fact that researchers move “back and forth between aspects of historical cases and alternative hypotheses that may help to account for those regularities,” thereby discovering “causal regularities that account for specifically defined historical processes or outcomes” (Skocpol 1984). “Learning from history” does not require scholars and those in power to be able to identify the causes that produce the causal powers, which lead to specific results. On the contrary, “learning from history” mainly intends to use previous historical experience to better understand the gains and losses of state governance, and such understanding is purpose-oriented, not pedantic. Regularity theorists do not require a specific category to produce a certain “causal power,” and even the connection between cause and effect is only derived from a “secret connexion” (Strawson 1989). Researchers of historical political science do not need to further explore what is behind the “secret connexion.” They only need to provide further ideological support for state governance in “learning from history” to complete the functional attribute of historical political science.

## Historical Explanation Based on the Logic and Evidences

Historical–causal narrative provides a new analysis principle, but it does not mean that researchers can indulge themselves in storytelling. Historical political science is not about interpreting historical or humanistic traditions, but about causal inference in the tradition of social sciences. Faced with various theories and viewpoints, researchers need to better understand the actual world based on sufficient dialogue. In the last part of this paper, I briefly introduce how to make causal inference using logic and evidence, in the historical narrative.

**Fig. 1:**
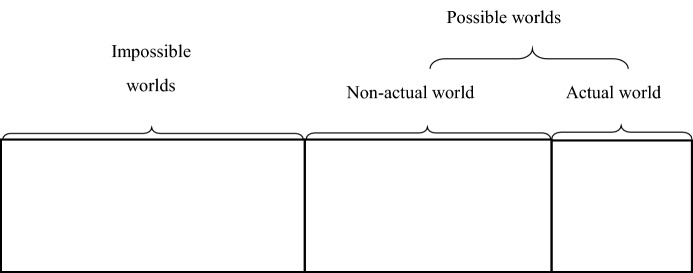
Types of possible worlds. Source: Raymond Bradley and Norman Swartz, Possible Worlds: An Introduction to Logic and Its Philosophy, Indianapolis: Hackett Publishing Company, 1988, p. 6

I draw on the idea of “possible worlds” in modal logic, based on the constructivist perspective (Bradley and Swartz 1979). According to David Lewis, A possible world is a spatio-temporal domain that does not violate a transcendental truth (Lewis 1986). In possible worlds, Africa may not be colonized, the Soviet Union may not collapse, there may be no COVID-19, and the Big Foot or the Loch Ness Monster may exist. However, among all the possible worlds, there is only one “actual world” in in which we inhabit. As opposed to the “actual world,” the “non-actual world” may not be the world we live in, but it is also part of the “possible worlds.” The “impossible worlds” are those that do not conform to logical common sense and law of nature and where, say, 1 + 1 = 3. Figure 1 shows the relationship between the possible worlds, the impossible worlds, the actual world, and the non-actual world. Researchers pay special attention to the phenomena in the possible worlds, because their existence is the product of the interaction between social reality and the human mind.

Although we live in the actual world, we are not fully confident that we truly understand this world. Various concepts have constructed many possible worlds. Regarding the nature of light, the hypothesis that “light is a wave” constitutes a possible world. Scientists in the nineteenth century believed, based on the technological and cognitive conditions available at the time, that the actual world we are in is exactly part of the possible world where the hypothesis is true. Later, however, Einstein’s study of the photoelectric effect shows that light is a photon, and the actual world we live in is no longer part of the possible world where the hypothesis that “light is a wave” is true; and with the development of quantum mechanics, scientists have discovered that light is both a wave and photon. Therefore, the actual world we live in is a possible world where wave-particle duality is true. We can see from this process that understanding the actual world is premised on the cultivation of observation, which can make people feel more confident about whether some possible worlds will become the actual world. In this process, observation turns a “possible world” into an “impossible one” (Barrenechea and Mahoney 2019).

**Fig. 2:**
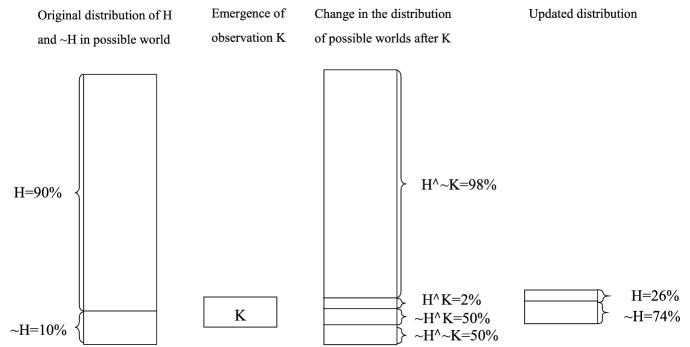
Set diagram illustration of the credibility of a theory by critical observation

In historical political analysis, logic and evidence can also be used to determine whether the actual world is a possible world, where a hypothesis is true. Due to the constructiveness of the social world, various concepts make it difficult for people to understand the actual world we live in. For example, the bourgeoisie or the middle class have long been regarded as the driving force of democracy. Therefore, many researchers are convinced that the actual world is a possible world where the hypothesis that “no bourgeoisie, no democracy” is true. However, researchers have falsified this myth when studying British political development since the nineteenth century; thus, separating the actual world from the possible world where the hypothesis is true (Yang 2009). As shown in Fig. 2, we present this process using set theory. According to popular theories, 90% of the actual world falls into the possible world where the hypothesis that “democracy is a product of the bourgeoisie” is true. Yet the emergence of a critical observation changes our perception: in the 1830s and 1840s, a large-scale charter movement took place in the United Kingdom. Workers demanded suffrage, which triggered fierce class conflicts and even suppression. If the hypothesis is true, the bourgeoisie, which supports democracy, should have never suppressed the workers asking for suffrage; or, if the bourgeoisie had achieved the mission of democracy, there would have been no such movement seeking suffrage. Therefore, the probability of the possible world where the hypothesis is true overlapping with the possible world where the observation appears, is very low; I would assume it to be 2%. Meanwhile, if the hypothesis is false, that is, “democracy is not achieved by the bourgeoisie,” then the historical context shown by the observation may or may not exist. We define the probability of the ambiguous overlapping as 50%. To sum up, the emergence of observance K excludes the possible world where 98% of the hypotheses are true and the possible world where 50% of the hypotheses are true false. The probability of the updated hypothesis being true is determined by the relative size of the remaining set. In the end, the possible world where a hypothesis is true is only about one-third of the possible world where a hypothesis is false. The emergence of observance reduces the probability of a hypothesis being true from 90 to 26%, posing a serious challenge to the truth of a theory.

**Fig. 3:**
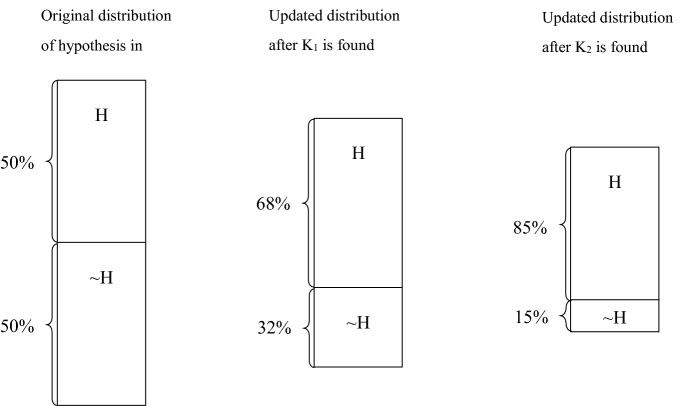
Cumulative observations and causal inference

I have shown above how “critical observation” is applied in causal inference and how it plays a decisive role in determining the truth of a theory or proposition. Researchers can use it to engage in dialogue with major theories. There is also “cumulative observation.” As shown in Fig. 3, such observation does not determine the truth of a hypothesis or theory, but as time goes by, the accumulation of cumulative observation can strengthen our confidence in judging hypotheses/theories. This type of observation is widely used in the study of gradual institutional changes (Mahoney and Thelen 2010). In historical political analysis, cumulative observation is particularly important because researchers need to observe how specific social existence is shaped and self-shaped in the ontological historical process. cumulative observations can embed causality into history, empowering the latter with a powerful vertical impetus.

We can, of course, call this impetus “causal mechanism.” The constructivist causality and the regularity theory of causality have provided causal mechanism with a meaning that distinguishes it from realism. Here, the “events” that constitute causal mechanism are analyzed in “sets.” The selection of “set” rather than “variable” provides process tracing with ontological attributes that makes it different from the variable-oriented research (Kreuzer 2016). Researchers of historical political science do not use evidence and logic to discover the substantive mechanisms with “causal power.” Instead, they use evidence to further understand the proportion of the actual world in the possible world where a hypothesis is true or false. This analysis method is based on Bayesian logic, which emphasizes that “our belief in the validity of a hypothesis is, after collecting evidence (posterior), equal to the probability of the evidence conditional on the hypothesis being true relative to other alternative hypotheses (likelihood), times the probability that a theory is true based on our prior knowledge”. The frequency school believes that the validity of causal inference is based on a large number of observations consistent with the hypothesis. But unlike this, the Bayesian school believes that a small amount (or maybe just one piece) of critical evidence can revise our previous views of the hypothesis/theory, that is, “what is important is not the number of pieces of evidence within a case that fit one explanation or another, but the likelihood of finding certain evidence if a theory is true versus the likelihood of finding this evidence if the alternative explanations are true” (Bennett 2009). With the increase of evidence, we can keep updating our understanding of the actual world in the process of “storytelling.”

Counterfactual thinking is fully utilized in historical political analysis to reveal the historical process in which social existence is formed. As mentioned above, the positivist tradition represented by quantitative research is based on counterfactual causality. However, “while counterfactuals are central to the very definition of causality in the quantitative tradition, the use of counterfactual analysis for causal inference drops out as option” (Goertz and Mahoney 2012). On the contrary, the way of thinking about possible worlds helps researchers better understand the actual world when they think about the non-actual world. For example, if China had not adhered to the leadership of the Communist Party of China (CPC) and adopted the democratic centralism regime, but instead had chosen the representative democracy of the West, what would have happened? A counterfactual analysis of nine developing countries with a population of over 100 million that embarked on different paths at the time of state-founding was done. In this, we find that only China, which adheres to the leadership of the CPC and implements the democratic centralism regime, has achieved economic development and social stability, while the remaining eight countries that implemented representative democracy are far inferior to China in terms of governance performance, even though some of them were ahead of China more than half a century ago (Yang 2016a, b). The story of “1 vs. 9” demonstrates through counterfactuals that democratic centralism is a necessary condition for China to become prosperous and strong. Re-recognizing the source of legitimacy and its knowledge base through comparison in history is an outstanding feature of historical political science that sets it apart from all other history-oriented social science research.

## Conclusions

After sorting out existing research and understanding the academic status quo, I am deeply aware of a “self-narrowing” tendency in the study of political science. One thing that impresses me most is that many interesting and meaningful issues are not studied by political researchers as they do not follow scientific norms. Take COVID-19 as an example. It could have been a topic of both academic significance and practical concern to explore the huge differences between China and the United States in their efforts to fight the pandemic. However, the research norms of scientism determine that comparison is infeasible for such a case as there are “too many variables” to ensure effective control. The purpose of this paper is to transcend such “common sense” that has been constructed in recent decades. According to Larry Laudan, science is, in essence, problem-solving activities. When evaluating the value of a theory, to what extent the theory gives appropriate explanations for major issues is more important than to what extent it fits the contemporary epistemological framework (Laudan 1978). The purpose of historical political science is to provide substantive enlightenment to the social structure in which we live.

Surely, this attempt will be questioned by many: positivists may think that historical political science will become more casual and lack scientific norms; and causal power theorists may even disagree with me as to the fundamental premise of whether causal mechanism is an “entity.” The vast differences between the social world and the natural world make it difficult for researchers to engage in political and social analysis under the cognitive model of naturalism. This paper intends to warn that the methodological bases on which many popular research methods depend are, in fact, extremely fragile. They are either abandoned in the scientific revolution or not suitable for analyzing macro-political and social phenomena. Blindly believing in the research methods formed by these views will result in a loss of sensitivity to the actual world. Based on the progress of contemporary philosophy of social sciences and the debate on research methods, I believe that the historical-causal narrative, which integrates the cognitive model of constructivism and the regularity theory of causality, not only provides a new analysis mode in the methodological debate, but also has the potential to provide a more solid methodological basis for historical political science.
